# Pluripotency of embryonic stem cells lacking clathrin-mediated endocytosis cannot be rescued by restoring cellular stiffness

**DOI:** 10.1074/jbc.AC120.014343

**Published:** 2021-01-13

**Authors:** Ridim D. Mote, Jyoti Yadav, Surya Bansi Singh, Mahak Tiwari, Shinde Laxmikant V, Shivprasad Patil, Deepa Subramanyam

**Affiliations:** 1 National Centre for Cell Science, SP Pune University Campus, Pune, India; 2 Babasaheb Ambedkar Marathwada University, Aurangabad, India; 3 Applied Parasitology Research Laboratory, Department of Zoology, JES College, Jalna, India; 4 Indian Institute of Science Education and Research, Pune, India; 5 Savitribai Phule Pune University, Pune, India

**Keywords:** clathrin heavy chain, embryonic stem cells, pluripotency, atomic force microscopy, stiffness, Young's modulus, actin cytoskeleton, reprogramming, actin, clathrin, embryonic stem cell, biophysics, cofilin

## Abstract

Mouse embryonic stem cells (mESCs) display unique mechanical properties, including low cellular stiffness in contrast to differentiated cells, which are stiffer. We have previously shown that mESCs lacking the clathrin heavy chain (*Cltc*), an essential component for clathrin-mediated endocytosis (CME), display a loss of pluripotency and an enhanced expression of differentiation markers. However, it is not known whether physical properties such as cellular stiffness also change upon loss of *Cltc*, similar to what is seen in differentiated cells, and if so, how these altered properties specifically impact pluripotency. Using atomic force microscopy (AFM), we demonstrate that mESCs lacking *Cltc* display higher Young's modulus, indicative of greater cellular stiffness, compared with WT mESCs. The increase in stiffness was accompanied by the presence of actin stress fibers and accumulation of the inactive, phosphorylated, actin-binding protein cofilin. Treatment of *Cltc* knockdown mESCs with actin polymerization inhibitors resulted in a decrease in the Young's modulus to values similar to those obtained with WT mESCs. However, a rescue in the expression profile of pluripotency factors was not obtained. Additionally, whereas WT mouse embryonic fibroblasts could be reprogrammed to a state of pluripotency, this was inhibited in the absence of *Cltc.* This indicates that the presence of active CME is essential for the pluripotency of embryonic stem cells. Additionally, whereas physical properties may serve as a simple readout of the cellular state, they may not always faithfully recapitulate the underlying molecular fate.

Early mammalian development is a complex process where different molecular mechanisms and signaling pathways regulate the choice of cell fate. Embryonic stem cells (ESCs) derived from the 3.5-day postcoitum blastocyst serve as a fantastic model system to study early cell fate decisions, as they have the ability to differentiate into all cell lineages (1). This property of ESCs is governed by numerous factors, including transcriptional networks involving molecules such as Oct4, Sox2, Nanog, etc. (2, 3), chromatin modifiers such as DNA methyltransferases and histone methyltransferases (4, 5, 6, 7), endocytic pathways (8, 9), and mechanical properties (10, 11, 12).

A growing body of work also implicates the process of intracellular trafficking and endocytosis in regulating the fate of embryonic stem cells (8, 9, 13, 14). Specific pathways, such as the clathrin-mediated endocytosis (CME) pathway (8), and endocytic proteins such as Asrij (13) are essential for maintaining the pluripotent state of ESCs, whereas other pathways, such as those involving caveolin, are largely absent in mouse ESCs (mESCs) (9). Furthermore, it has been demonstrated that the expression of endocytic genes is altered during human somatic cell reprogramming (14). Endocytosis itself is also affected by mechanical properties of the cell, such as membrane stiffness, with increasing stiffness resulting in an inhibition of vesicular trafficking (15, 16, 17). The effect of stiffness on endocytosis can be countered by an active involvement of actin at endocytic sites (18, 19).

Mechanical properties have also been shown to regulate the pluripotency of ESCs, with cell stiffness or elasticity being one of the major mechanical parameters governing cell fate. Atomic force microscopy (AFM) analysis carried out on pluripotent mESCs and early differentiating mESCs showed a 2–3-fold increase in the elastic modulus of early differentiating mESCs compared with naive mESCs (11), with modulation of the actin cytoskeleton resulting in a change in stiffness (20, 21).

Study of the actin cytoskeleton in mESCs has revealed it to be a low-density meshwork, possessing larger pore size and independent of myosin, compared with differentiated cells (21). Inhibition of actin polymerization in mESCs has also been shown to result in a decrease in differentiation toward the mesodermal lineage coupled with an increase in differentiation toward the endodermal lineage (22). Naive mESCs had few radial actin structures, whereas primed mESCs showed an increase in stiffness with more actin bundles near the cell periphery (23). Signaling molecules such as β-catenin have also been shown to be involved in regulating a change in cytoskeletal organization, membrane tension, and subsequently endocytosis during ESC differentiation (24). Whereas these studies reveal that the differentiation of ESCs is a tightly regulated process requiring an intricate interplay between mechanical, cytoskeletal, and transcriptional factors, a clear hierarchy in the role of these factors has yet to emerge.

Previous work from our laboratory has demonstrated an important role for the clathrin heavy chain (*Cltc*) in maintaining the pluripotent state of mESCs. CLTC is an integral part of the clathrin coat in CME. Knockdown (KD) of *Cltc* in mESCs resulted in a loss of CME and reduction in the pluripotency of mESCs, resulting in an initiation of differentiation (8). Previous studies have demonstrated that differentiating mESCs have a high stiffness and higher Young's modulus compared with pluripotent mESCs (11). Additionally, a transition from the naive state to the primed state is also accompanied by a reorganization of the actin cytoskeleton and an alteration in viscoelastic properties (23). As a follow-up to our previous study (8), we asked whether the loss of CME was also accompanied by changes in the stiffness of these cells, similar to other differentiating cells, and whether the actin cytoskeleton played a role under these conditions.

Using AFM, we measure for the first time the Young's modulus of live mESCs lacking *Cltc* plated on Matrigel using a spherical bead attached to a cantilever. We show that cells lacking *Cltc* have a higher Young's modulus compared with WT mESCs. We further demonstrate that mESCs lacking *Cltc* display an enhancement in the presence of actin stress fibers in cells, which are largely absent in WT mESCs. This is also accompanied by an elevated expression of the inactive, phosphorylated form of the actin-depolymerizing protein, cofilin, resulting in the presence of stable actin filaments. Treatment of *Cltc* KD mESCs with the actin polymerization inhibitors, latrunculin A and cytochalasin D, resulted in a rescue of cellular stiffness, with cells reverting to a state closer to WT mESCs with respect to mechanical properties. However, the expression profile of pluripotency factors was not rescued, and continued to resemble that of a differentiating cell, indicating that alterations in the actin cytoskeleton may not be able to rescue pluripotency in the absence of *Cltc* and hinting at a role for active CME in this process. Reprogramming of somatic cells was also inhibited in the absence of *Cltc.* Together, these results suggest that the pluripotent state is an amalgamation of both mechanical and molecular properties, which function together to influence the state of a cell. Additionally, a change in a single readout may not be sufficient to completely predict or alter the state of a cell and may be dependent on the co-existence or coordinated function of other factors.

## Results

### Cltc KD results in increased cell stiffness and reorganization of the actin cytoskeleton in mESCs

CME is a type of vesicular transport in which receptors are internalized into intracellular structures called endosomes with the help of the coat protein, clathrin (25). We have previously shown that KD of *Cltc* in mESCs resulted in a decreased expression of pluripotency markers and an increased expression of differentiation markers of all three germ layers (8). However, the mechanical properties of mESCs under these conditions remained to be interrogated. *Cltc* was knocked down in mESCs using two shRNAs (shCltc1 and shCltc3) (Table S1), resulting in a significant decrease in protein levels of CLTC compared with cells infected with the lentivirus expressing the scrambled shRNA (Fig. S1, A and B) (Table S3), and was used for all further experiments.

A tipless cantilever with stiffness of ∼0.07 newton/m, with a spherical glass bead of diameter 5 μm attached to the end, was used for all AFM measurements (Figs. S1C and S^2^) (see “Materials and methods”). In AFM investigations, the spatial resolution depends on sharpness of the tip. Sharp pyramidal tips are generally used for measurements of mechanical response at a subcellular level on components such as the cytoplasm and nucleus, combined with high-resolution imaging. Tipless cantilevers with a spherical bead attached to one end (Fig. S1C) are used to measure deformations and resulting stress of inhomogeneous surfaces and are used to investigate the response of the cell as a whole (26). The spherical tips also provide more contact area for measurement and prevent cell damage by avoiding membrane rupture.

The inherent approximation in all of these studies is that the glass-tip contact is nondeformable and hence has infinite stiffness. As a result, all of the reported values of stiffness using AFM may have a systematic error in their absolute values. However, this assumption is largely valid for measurements on cells and tissues for which the Young's modulus is on the order of few kPa. The supporting material shows representative force curves on cells with respect to glass and Matrigel (Fig. S2).

To measure the Young's modulus of Matrigel, a 3 × 3-μm area was selected, and measurements were taken over a (10 × 10) grid. The Young's modulus of Matrigel was found to be in the range of 2–3 kPa. These values are higher than those reported earlier (10, 27), and can be attributed to differences in protocols adopted for preparing the gel and also the size of the microspheres used in the AFM measurement.

**Figure 1:**
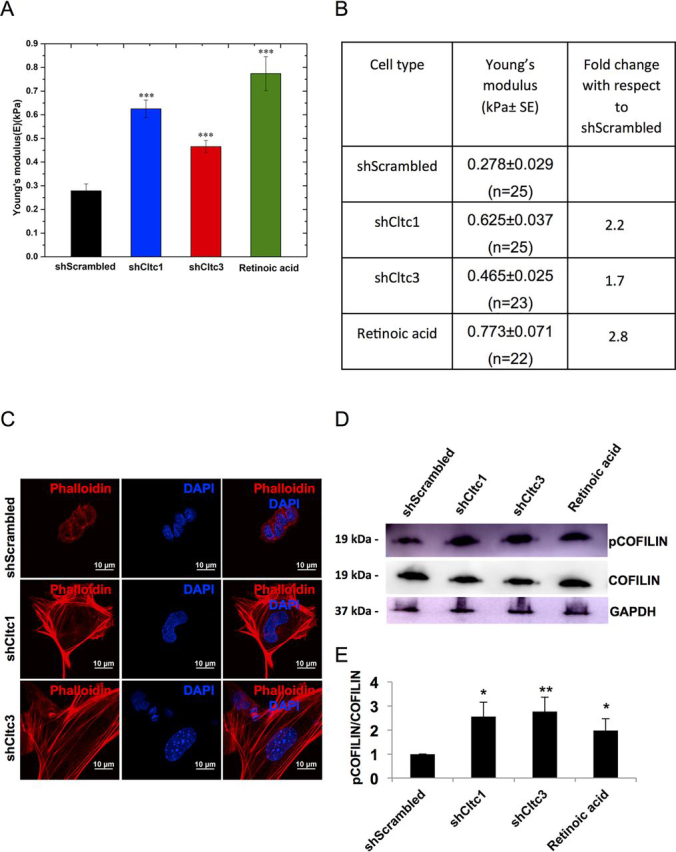
***Cltc* KD results in increased cell stiffness and reorganization of the actin cytoskeleton in mESCs.***A*, Young's modulus (*E*) of shScrambled-, shCltc1-, shCltc3-, and RA-treated mESCs. ***, *p* < 0.0001 by Student's *t* test. *Error bars*, S.E. over 22–25 cells with 100 measurements on each cell with a 3 × 3-μm grid. ∼2000 force curves were collected for each condition. *B*, the table shows the apparent Young's modulus (*E*) of cells under the mentioned conditions (*n* denotes the number of cells analyzed). *C*, representative confocal micrographs showing actin filaments stained with phalloidin in shScrambled, shCltc1, and shCltc3 mESCs. *Scale bar*, 10 μm (*n* = 15). *D*, Western blotting showing expression of pCOFILIN, COFILIN, and GAPDH in shScrambled-, shCltc1-, shCltc3-, and RA-treated mESCs. *E*, quantitation of pCOFILIN/COFILIN levels from Western blots. *Error bars*, S.D. for experiments (*n* = 3). *, *p* < 0.05; **, *p* < 0.01 by Student's *t* test.

Young's modulus (*E*) for mESCs expressing scrambled shRNA was 0.278 ± 0.029 kPa, whereas *E* for cells expressing shCltc1 was 0.625 ± 0.037 kPa, and *E* for cells expressing shCltc3 was 0.465 ± 0.025 kPa, indicating higher stiffness in *Cltc* KD mESCs (Fig. 1, *A* and *B*). The raw data clearly revealed a variation in relative stiffness of cells under different knockdown conditions (Fig. S2). The stiffness difference between shCltc1 and shCltc3 was also statistically significant with a confidence level of 95% (*p* = 0.02) and correlated with the degree of *Cltc* KD in these two conditions (Fig. S1, A and B). Measurements were also made from mESCs treated with retinoic acid (RA) for 48 h to induce differentiation (Young's modulus 0.773 ± 0.071 kPa), indicating greater stiffness (Fig. 1, *A* and *B*). Our results demonstrate that the Young's modulus increased by 2.2-fold in shCltc1, 1.7-fold in shCltc3, and 2.8-fold in RA-treated mESCs compared with shScrambled mESCs, indicating an increase in cellular stiffness upon loss of CME and/or subsequent differentiation.

The actin cytoskeleton is one of the major regulators of cellular stiffness, as it provides mechanical stability to adherent cells (21, 28). Phalloidin staining revealed that actin stress fibers were predominantly present in *Cltc* KD mESCs compared with shScrambled-treated cells (Fig. 1*C*). Actin filaments are dynamic structures and are involved in multiple cellular processes such as cell migration, cell division, endocytosis, etc. (19, 29). Actin filament dynamics are regulated by a number of actin-binding proteins. Among the actin-binding proteins, actin-depolymerizing factors or destrin and cofilin family proteins are involved in the depolymerization of actin filaments (29, 30, 31). LIM kinases (LIMK1 and LIMK2) and related testicular protein kinases (TESK1 and TESK2 in mammals) are known to inhibit the activity of cofilin. These kinases phosphorylate cofilin at Ser-3 resulting in an inhibition of cofilin's actin-depolymerizing activity (29, 30). Hence, we investigated the status of cofilin phosphorylation in *Cltc* KD mESCs. Phosphorylation of cofilin at the Ser-3 residue was higher in *Cltc* KD mESCs compared with scrambled shRNA-treated mESCs (Fig. 1, *D* and *E*), indicating a high degree of inactivation and consistent with an increase of actin stress fibers in *Cltc* KD mESCs (Fig. 1*C*). Together, these results indicate that the loss of *Cltc* results in an altered organization of the actin cytoskeleton.

### F-actin–depolymerizing agents reduce the stiffness of Cltc-deficient mESCs

**Figure 2:**
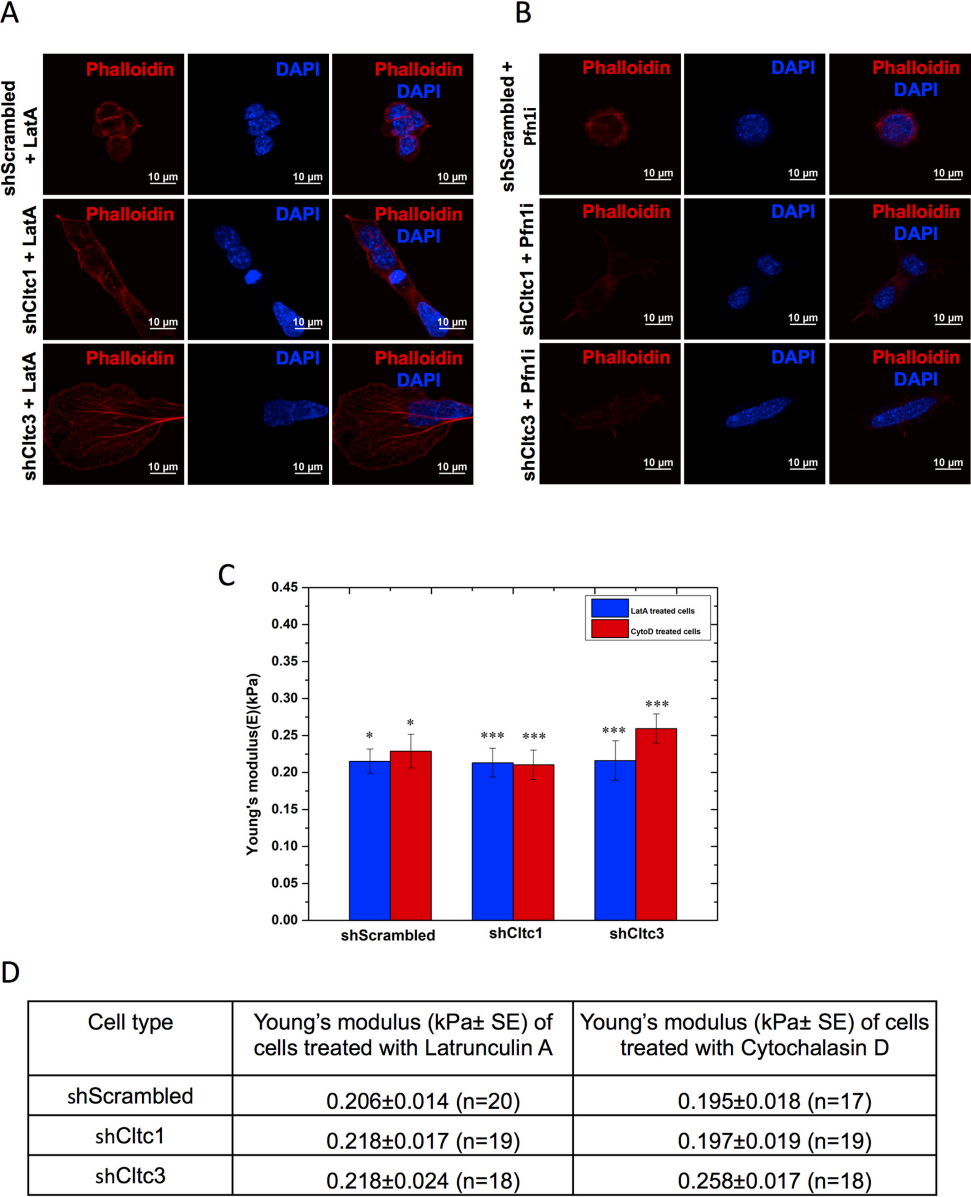
**F-actin–depolymerizing agents reduce the stiffness of *Cltc* KD mESCs.***A* and *B*, representative confocal micrographs showing actin filaments in shScrambled, shCltc1, and shCltc3 mESCs treated with LatA (0.1 μm) for 12 h (*A*) and under Pfn1 KD (Pfn1i) conditions and stained using phalloidin (*B*). *Scale bar*, 10 μm (*n* = 15). *C–E*, bar graph (*C*) and table (*D*) showing Young's modulus (*E*) of shScrambled-, shCltc1-, and shCltc3-treated mESCs under the mentioned conditions. Significance was calculated with respect to the corresponding untreated samples. *, *p* < 0.05; ***, *p* < 0.0001 by Student's *t* test. *Error bars*, S.E. over 17–20 cells with 100 measurements on each cell (*n* denotes the number of cells analyzed).

Treatment of *Cltc* KD mESCs with an inhibitor of actin polymerization, latrunculin A (LatA) or KD of the actin polymerizing protein, Profilin1 (Pfn1), resulted in a discernible loss of actin fibers (Fig. 2, *A* and *B*), indicating that the reorganization of the actin cytoskeleton in *Cltc* KD can be reversed by the action of LatA or KD of Pfn1. To further validate whether the actin cytoskeleton was indeed the major regulator of cellular stiffness and differentiation observed in *Cltc-*deficient mESCs, we treated shScrambled and shCltc mESCs with inhibitors of actin polymerization, LatA and cytochalasin D (CytoD). Upon treatment with LatA, the Young's modulus for shScrambled mESCs was 0.206 ± 0.014 kPa (Fig. 2, *C* and *D*), indicating a decrease in stiffness compared with untreated shScrambled mESCs (Fig. 1, *A* and *B*) and similar to what has been reported previously (21). A reduction in the Young's modulus was also observed for shCltc1 (0.218 ± 0.017 kPa) and for shCltc3 (0.218 ± 0.024 kPa) mESCs upon LatA treatment (Fig. 2, *C* and *D*), consistent with our observation of a decrease in stress fibers (Fig. 2, *A* and *B*). Similarly, upon treatment with CytoD, the Young's modulus for shCltc1 and shCltc3 reduced to 0.197 ± 0.019 and 0.258 ± 0.017 kPa, respectively, and was comparable with that of shScrambled mESCs (0.195 ± 0.018 kPa) (Fig. 2, *C* and *D*).

### Treatment of Cltc KD mESCs with F-actin–depolymerizing agents does not rescue the expression of pluripotency and differentiation markers

**Figure 3:**
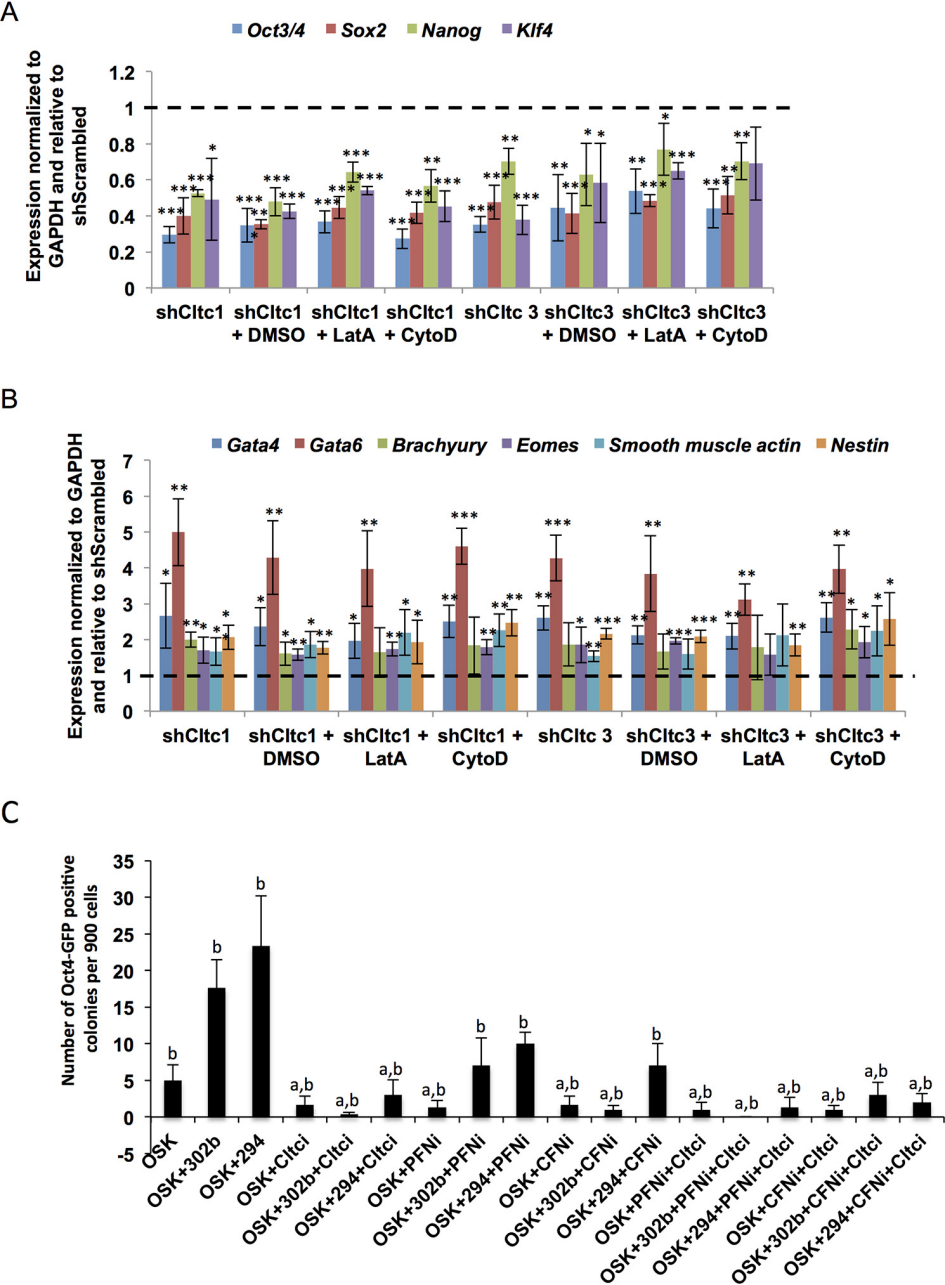
**CLTC is essential for restoration of pluripotency in the context of actin modulation in mESCs.***A* and *B*, RT-qPCR analysis of pluripotency markers (*A*) and differentiation markers (*B*) in shScrambled, shCltc1, and shCltc3 upon treatment with either DMSO, LatA (0.1 μm), or CytoD (0.2 μm) for 12 h. The bar graph shows the expression of markers in mESCs under the indicated conditions relative to the relevant shScrambled control. Control is shown as a *dotted line* at 1. For all experiments, *error bars* represent S.D. (*n* = 3). *, *p* < 0.05; **, *p* < 0.01; ***, *p* < 0.001 by Student's *t* test. *C*, graph showing the number of Oct4-GFP–positive colonies obtained at day 14 of reprogramming per 900 Oct4-GFP MEFs. Significance was determined by one-way analysis of variance followed by post hoc Tukey–Kramer test. *a*, *p* value <0.05 compared with OSK + miR-302b; *b*, *p* value <0.05 compared with OSK + miR-294.

Because treatment of *Cltc* KD mESCs with LatA or CytoD resulted in a decrease in stress fibers and cellular stiffness to levels similar to those of pluripotent WT mESCs, we next asked whether the expression of pluripotency and differentiation markers were also restored to levels seen in WT mESCs. *Cltc* KD mESCs showed decreased expression of pluripotency markers and increased expression of differentiation markers irrespective of plating on either gelatin or Matrigel (Fig. 3 (*A* and *B*) and Fig. S1 (D–G)) (Table S2) and as reported previously (8). However, treatment with LatA or CytoD over different concentrations and time periods did not result in any significant change or rescue in the expression of pluripotency and differentiation markers (Fig. 3 (*A* and *B*) and Figs. S1G, S3, and S4), suggesting that this may not be possible in the absence of active CME. To determine whether the temporal order of actin modulation relative to *Cltc* KD could rescue the differentiation of mESCs, KD of Profilin1 (Pfn1), an actin monomer–binding protein involved in actin filament elongation (31), was combined with *Cltc* KD. *Cltc* KD mESCs maintained a loss of pluripotency marker expression irrespective of the sequence of actin modulation (Fig. S5).

To further determine whether CLTC and actin-modulating proteins also influenced the acquisition of pluripotency, we reprogrammed somatic cells to iPSCs. Reprogramming of somatic cells to iPSCs is a complex process involving extensive cytoskeletal remodeling (14, 32, 33). ESCC microRNAs such as miR-302b or miR-294 are known to significantly enhance the reprogramming of somatic cells and provide a better experimental condition to assess the influence of enhancers or inhibitors of reprogramming (14, 34). However, KD of *Cltc* significantly decreased the reprogramming efficiency in mouse embryo fibroblasts (MEFs) infected with retroviruses encoding Oct4, Sox, and Klf4 and transfected with miR-302b or miR-294 (Fig. 3*C*). Sakurai *et al.* (33) have previously reported that the knockdown of TESK1, a regulator of cofilin, enhanced reprogramming of somatic cells. Analysis of the role of actin-binding proteins (ABPs) cofilin and Profilin1 in our assay revealed that the KD of these proteins individually did not influence reprogramming (Fig. 3*C*). In fact, knockdown of these proteins also resulted in a decrease in reprogramming efficiency in the context of enhancers such as miR-294, hinting at a complex interplay between actin reorganization and CME to achieve a pluripotent state (Fig. 3*C*). Reprogramming involves a temporal progression from a mesenchymal to an epithelial state (35, 36), and it is possible that the involvement of actin-reorganizing proteins may be required in a stage-specific manner. Together these results indicate that whereas the actin cytoskeleton is largely involved in the regulation of cellular stiffness, its modulation may not result in an alteration of the pluripotency network under conditions where CME is not functional.

## Discussion

We have recently demonstrated that the loss of CME results in decreased expression of pluripotency factors in mESCs, accompanied by an increase in the expression of differentiation markers (8). Here, we show that mESCs lacking CME display greater stiffness or Young's modulus (*E*), as determined by AFM measurements (Fig. 1). Previous studies have also reported that the stiffness of early differentiating ESCs is higher, compared with undifferentiated ESCs, as determined by AFM (11). Our data also demonstrate that mESCs lacking *Cltc* display a reorganized cytoskeleton, which is largely responsible for the higher mechanical stiffness compared with WT cells. However, a rescue in stiffness of the *Cltc* KD ESCs through modulation of the actin cytoskeleton did not result in a rescue of expression of pluripotency markers (Figure 1, Figure 2, Figure 3).

ESCs are a potential source of cells for regenerative medicine, which have applications for therapeutic purposes in a variety of diseases. Treatment for these diseases requires efficient protocols for terminal differentiation and ultimately purification of these cells. Classically, researchers have concentrated on a handful of markers to decide whether a cell is pluripotent or has undergone differentiation to give rise to a specialized cell type. More recently, it has become increasingly obvious that other factors contribute by varying degrees to the pluripotent state of a stem cell. These include endocytic pathways and proteins, mechanical properties, and transcription factors and epigenetic modifiers. Recent reports describe using mechanical phenotyping as a label-free and efficient technique for large-scale purification of cells (37). However, a better understanding of the interplay between mechanical properties and expression of stem cell–specific transcription factors is required before mechanical phenotyping can be used for large-scale purification of cells.

Cellular stiffness has been shown to be accompanied by an increased polymerization of the actin cytoskeleton. Studies using superresolution microscopy have demonstrated that mESCs are softer and have a low-density meshwork of F-actin, with large pore sizes, compared with stiffer, differentiated cells (21). Stem cells transitioning from the naive to the primed state of pluripotency also display a reorganization of the actin cytoskeleton (23). Our data show that the loss of CME in mESCs results in the presence of stress fibers, which are largely absent in WT mESCs (Fig. 1). These cells also display an enhanced level of the inactive, phosphorylated form of the actin-binding protein cofilin, in *Cltc* KD and RA-treated ESCs. The increase in stiffness of mESCs upon KD of *Cltc* could be rescued by treatment with inhibitors of actin polymerization, LatA or CytoD. Previous reports also demonstrated that knocking down the actin-capping protein Capzb resulted in increased stiffness of stem cells, whereas treatment with actin polymerization inhibitors reduced the stiffness. These effects were independent of myosin activity and were dependent on the activity of formin and Arp2/3 (21).

However, whereas these studies demonstrated that the actin cytoskeleton and properties associated with it changed during the differentiation of stem cells, they did not address the question of whether reverting the state of the actin cytoskeleton could re-establish the pluripotent state. In other words, can the state of the actin cytoskeleton play a dominant role in defining the fate of a cell? We observed that although treatment of *Cltc* KD mESCs with actin polymerization inhibitors reduced their stiffness to levels similar to WT mESCs, such treatments did not result in a rescue in expression of pluripotency markers. Previous reports suggest that modulation of the phosphorylation status of actin-depolymerizing factors, such as cofilin, can regulate the reprogramming of somatic cells (33). In contrast, Xia *et al.* (21) report that a depletion of cofilin did not result in an alteration of the cortical architecture of ESCs. In our hands, individual KD of ABPs did not significantly change the efficiency of reprogramming (Fig. 3*C*). Increase in reprogramming efficiency by the action of miR-294 or miR-302b was, however, significantly inhibited by the KD of *Cltc* or ABPs, suggesting that the temporal control of active intracellular transport through the clathrin pathway and rearrangement of the cytoskeleton may be critical requirements for attaining pluripotency. An initiation of CME may be essential for the transport of molecules that ultimately regulate the pluripotency network of a stem cell. Our results also suggest that a rescue of mechanical properties need not necessarily always reflect a change in the transcriptional network of ESCs. We would also like to postulate that measurement of individual readouts of a stem cell may result in inaccurate conclusions regarding its actual state if the underlying molecular networks and cellular processes are not taken into consideration. Furthermore, this may also suggest that an inherent hierarchy may exist with respect to specific events that dictate when a cell achieves pluripotency. A pluripotent ESC is thus the result of a complex interplay between many different molecular, intracellular, and mechanical players, and a single readout may not be sufficient to definitively determine its state.

## Experimental procedures

### Mouse embryonic stem cell culture

V6.5 mESCs were cultured on tissue culture plates (Corning) coated with 0.2% gelatin in knockout Dulbecco's modified Eagle's medium supplemented with 15% fetal bovine serum, 0.1 mm β-mercaptoethanol, 2 mm l-glutamine, 0.1 mm nonessential amino acids, 5000 units/ml penicillin/streptomycin, and 1000 units/ml LIF (ESC medium). Cells were passaged using trypsin, every 3 days.

### Cell mechanics using AFM

A thin layer of Matrigel was coated on 22-mm glass coverslips. To measure the Young's modulus of Matrigel, a 3 × 3-μm area was selected, and measurements were taken over a (10 × 10) grid. The Young's modulus of Matrigel was found to be in the range of 2–3 kPa. These values are higher than those reported earlier (10, 27) and can be attributed to the differences in protocols adopted for preparing the gel and also the size of the microspheres used in the AFM measurement. The cells were deformed under a glass microsphere attached to the cantilever, which is of similar size as the cell. This deformation (indentation) was less than 1 μm, as seen in Fig. S2. The cell thickness when plated on Matrigel was about 3–5 μm. Because the Young's modulus of the cells was below 1 kPa, this deformation was attained with forces less than 1 nanonewton, avoiding too much loading of the cells. For each cell, a 3 × 3-μm area was selected, and 100 force curves were taken in a 10 × 10 grid at different locations on a single cell. All measurements were done on live cells only. Indentation studies were performed on more than 20 control (undifferentiated) mESCs and treated cells (*Cltc* KD or RA-treated). The approach velocity was 2 μm/s with a sampling rate of 2048 data points/s.

### AFM analysis

Assuming the glass-glass contact to be infinitely stiff compared with the glass-cell contact—a reasonable assumption because it is 10,000 times stiffer—the slope of the curve in the contact region for glass is 1, implying no deformation (Figs. S1C and S^2^). The slope of the curve on cells is much less, suggesting a certain amount of deformation. We used glass-glass contact for calibration of deflection sensitivity, and the subtraction of cantilever deflection from the push given by the piezo extension yields deformation in the tissue. The force is calculated by multiplying the cantilever deflections by its stiffness. The force *versus* deformation curve is then fitted with the Hertz model,

(Eq. 1) $$F=\frac{E}{\left(1-v^{2}\right)}\left(\left({(a}^{2}+R_{S}^{2})/2\right)ln\left(\frac{R_{S}+a}{R_{S}-a}\right)-aR_{S}\right)$$

(Eq. 2) $$\text{δ}\text{=}(\text{a/2})\text{ln}\left(\left(R_{S}+a)/(R_{S}-a\right)\right)$$

where *F* is measured by the cantilever possessing the bead, which is pressed against the cell. *R* is the bead radius, δ is the deformation in the cell, *E* is the Young's modulus, and *v* is the Poisson ratio.

## Data availability

All data are included in the article.

10.13039/501100009053The Wellcome Trust DBT India Alliance (India Alliance) (IA/I/12/1/500507) to Deepa Subramanyam
10.13039/501100009053The Wellcome Trust DBT India Alliance (India Alliance) (500172/Z/09/Z) to Shivprasad Patil
Department of Biotechnology, India (BT/PR30450/MED/31/399/2018) to Deepa Subramanyam
