# Supplementary material for: Pluripotency of embryonic stem cells lacking clathrin-mediated endocytosis cannot be rescued by restoring cellular stiffness
Source: J Biol Chem. 2021 Jan 13;295(49):16888–96. doi: 10.1074/jbc.AC120.014343 (PMC7864080; doi:10.1074/jbc.AC120.014343)
Supplement: Supplementary file 1 [file mmc1.pdf]

Supplementary Information: Supplementary information contains supplementary methods, figures and figure legends.

Supplementary Methods:

Lentivirus packaging and infection: Lentiviral vectors pLKO.1-shScrambled, pLKO.1-shCltc1/ shCltc3 were individually cotransfected with psPAX2 (ADDGENE NO. 12260), pMD2.G (ADDGENE NO. 12259) in HEK293T cells using FuGENE HD (Promega E2311). pLKO.1-shCltc1, and shCltc3 were obtained from the shRNA Resource Centre, Indian Institute of Science, Bangalore. Viral supernatants were harvested and used for infection 60 hours post transfection. 1.5×10^5 mESCs were infected with packaged lentiviruses with polybrene in suspension, followed by plating on gelatin-coated plates (Corning CLS3516). The following day, lentivirus-containing media was removed and ESC media was added to the cells. 24 hours later, cells were trypsinized and 3×10^4 cells were plated on matrigel (Corning 356234) coated 22mm coverslips in ESC media containing puromycin (1ug/ml). AFM experiments were performed on live cells 12 hours post plating.

Inhibitor treatment: 5×10^4 mESCs of shScrambled, shCltc1 and shCltc3 were plated on 0.2% gelatin coated 24 well plates. After 12hrs of plating, cells were treated with the following inhibitors of actin polymerization: Latrunculin A (LatA) (Sigma L5163), and Cytochalasin D (CytoD) (Sigma C8273) at the specified concentrations and duration. After 12 hrs, samples were processed for RNA isolation. Retinoic acid (RA) was added to mECSs at a concentration of 10^{-7}M in the absence of LIF.

RNA isolation and Real-time PCR: Total RNA was isolated from mESCs using TRIzol and quantified using a Nanodrop spectrophotometer. 1ug of total RNA was used to synthesize complementary DNA (cDNA) using Verso cDNA synthesis Kit from Thermo Scientific (#AB-1453/A). Gene-specific primers for RT-qPCR were designed using IDT software (sequences provided in Supp. Table 2). ABI Power SYBR Green PCR master mix was used for quantitative RT–PCR reactions. ABI qPCR system, 7900 HT was used to perform quantitative RT–PCR reactions.

Western blotting: Protein lysates were prepared from cells using RIPA buffer containing proteinase inhibitors and PMSF. Protein concentration was determined using Bradford reagent. Equal concentration of total protein lysates were subjected to SDS-PAGE electrophoresis followed by transfer to PVDF membrane. After transfer, the membrane was blocked in 5% BSA. Post blocking, the membrane was incubated with the appropriate primary antibody at 4°C overnight with gentle rocking. Next day the blot was washed twice for 10 min using Tris-buffered saline (1X TBS) containing 0.1% Tween-20 (TBS-T). Membranes were incubated with an HRP-conjugated secondary antibody (1:1000) for 1 hour at room temperature. Thermo Scientific SuperSignal West Femto Maximum Sensitivity Substrate (Cat. no. 34095) reagent was added to the membranes, and images were captured post-exposure using a chemi-doc system (GE Healthcare, AI600). Western blot images were quantified using ImageJ software.

Immunocytochemistry and imaging: 2×10^4 mESCs were cultured on glass coverslips coated with matrigel in a 24 well plate. Following day, cells were washed with PBS, fixed with 4% paraformaldehyde at room temperature for 20 min, and permeabilized with 0.1% Triton X-100 in PBS for 5 min. After blocking in 5% bovine serum albumin in PBS for 1 h, cells were incubated with Phalloidin Alexa Fluor 568 (1:400) for 30 min. After 1 wash with 1X PBS, nuclei were stained with DAPI. After 2 washes, coverslips were mounted onto glass slides using VECTASHIELD (Vector Laboratories). Images were acquired using a Nikon Ti Eclipse confocal microscope with Plan Apo 60x oil objective (NA 1.4) at 512 × 512 pixels and 8 bit resolution.

Bead attachment on cantilever: A tipless cantilever with stiffness of ~0.07 N/m was used for AFM measurement. A spherical glass bead with diameter 5μm was attached to the end of the cantilever. The attachment of the bead on the cantilever was done using micromanipulation available with AFM. An approximate equal amount of araldite adhesive (locally sourced) and hardener glue was taken and mixed properly. With the help of a sharp toothpick, small dots were made on a glass slide. Using the servo control of AFM, the cantilever was brought down on the araldite glue. Since the bead was of very small size, a very small amount of glue was picked up on the cantilever, and the cantilever was again lowered down on the glass slide in order to remove the excess glue. After that the cantilever was lowered down onto the bead. Cantilever was maintained under positive load and after 5 minutes, the cantilever was pulled back along with the bead. Before measurements, the cantilever was calibrated each time on the glass coverslip.

Reprogramming: For reprogramming, on Day 0, 900 Oct4-GFP mouse embryonic fibroblasts (MEFs) cells were plated per well in 96 well plates coated with 0.2% gelatin in DMEM supplemented with 10% fetal bovine serum, 0.1 mM beta mercaptoethanol, 2mM L-glutamine, 0.1 mM nonessential amino acids, 5000 U/ml penicillin/streptomycin. On day 1, cells were infected with amphotropic retroviruses encoding for Oct4, Sox2 & Klf4. On day 3, cells were transfected with miR-302b or miR-294 mimics at a final concentration of 50nM, and Cltc, Pfn1, Cfn or non-targeting (NT) siRNAs at a final concentration of 50ng. On day 4, media was changed to ES media and changed daily. On day 7, cells were retransfected. From day 8, onwards cells were cultured in KO DMEM supplemented with 15% Knockout serum replacement (KOSR) media containing LIF. Reprogrammed colonies were counted on Day 14 by observing ESC morphology and activation of GFP.

Supplementary Figure Legends:

Supp. Fig. 1: Characterization of *Cltc* KD in mESCs:

A) Western blot showing expression, and B) quantitation of CLTC and TUBULIN in shScrambled, shCltc1, shCltc3 mESCs. C) Schematic of an AFM cantilever to measure cell stiffness. The inset shows an image of the cantilever attached with a 5 μm bead. RT-qPCR analysis of (D) pluripotency markers, and (E) differentiation markers in shCltc1 on gelatin and matrigel relative to the relevant shScrambled control. F) RT-qPCR analysis showing KD of *Cltc* mRNA in shCltc1 under same conditions as D) and E). G) RT-qPCR analysis showing KD of Cltc mRNA in shCltc1 and shCltc3, under indicated conditions relative to the relevant shScrambled control. Control is shown as a dotted line at 1. For all experiments, error bars represent mean ± S.D for experiments in triplicates (N = 3). *$p < 0.05$; **$p < 0.01$, ***$p < 0.001$ by Students T-test.

Supp. Fig. 2: Representative force curves on samples:

Representative force curves on all samples plotted together. The cells are deformed under a glass microsphere attached to the cantilever which is of similar size as the cell. This deformation (indentation) is less than 1 μm as seen in the figure. The cell thickness when it is plated on matrigel is about 3-5 μm. This data is used to estimate the Young's modulus reported in Fig. 1 and Fig. 2. Raw data clearly reveals variation in stiffness in mESCs after various treatments and KDs. Glass cover-slip is a reference and the tip-glass contact is assumed to be non-deforming.

Supp. Fig. 3: LatA (0.05uM) and CytoD (0.1uM) treatments are unable to rescue pluripotency marker expression in *Cltc* KD mESCs

RT-qPCR analysis of A) pluripotency markers and B) differentiation markers in shScrambled, shCltc1 and shCltc3 upon treatment with either DMSO or LatA (0.05uM) or CytoD (0.1uM) for 12hrs. Bar graph shows the expression of A) pluripotency and B) differentiation markers in mESCs under indicated conditions relative to the relevant shScrambled control. Control is shown as a dotted line at 1. C) RT-qPCR analysis showing KD of *Cltc* mRNA in shCltc1 and shCltc3 under same conditions as A) and B).

For all experiments, error bars represent mean ± S.D for experiments in triplicates (N = 3). *p < 0.05; **p < 0.01, ***p < 0.001 by Students T-test.

Supp. Fig. 4: LatA (0.05uM) and CytoD (0.1uM) treatment for 24hrs are unable to rescue pluripotency marker expression in *Cltc* KD mESCs
RT-qPCR analysis of A) pluripotency markers and B) differentiation markers in shScrambled, shCltc1 and shCltc3 upon treatment with either DMSO or LatA (0.05uM) or CytoD (0.1uM) for 24hrs. Bar graph showing the expression of A) pluripotency and B) differentiation markers in mESCs under indicated conditions relative to the relevant shScrambled control. Control is shown as a dotted line at 1. C) RT-qPCR analysis showing KD of Cltc mRNA in shCltc1 and shCltc3 under same conditions as A) and B). Control is shown as a dotted line at 1. For all experiments, error bars represent mean ± S.D for experiments in triplicates (N = 3). *p < 0.05; **p < 0.01, ***p < 0.001 by Students T-test.

Supp. Fig. 5: Pfn1 KD cannot rescue the expression of pluripotency markers in *Cltc* KD mESCs
A) RT-qPCR analysis of pluripotency markers under different KD conditions. Bar graph showing the expression of pluripotency markers in mESCs under indicated conditions. D1 depicts day1 of transfection and D2 depicts day2 of transfection. Control is shown as a dotted line at 1. B & C) RT-qPCR analysis showing KD of *Cltc* and *Pfn1* mRNA under indicated conditions relative to non-targeting (NT) siRNA control. Control is shown as a dotted line at 1. For all experiments, error bars represent mean ± S.D for experiments in triplicates (N = 3). *p < 0.05; **p < 0.01, ***p < 0.001 by Students T-test.

Supplemental Table 1: Table showing *Cltc* shRNA sequences.
Supplemental Table 2: List of primers used in this study.
Supplemental Table 3: List of antibodies used in this study.

Supplementary Figure 1

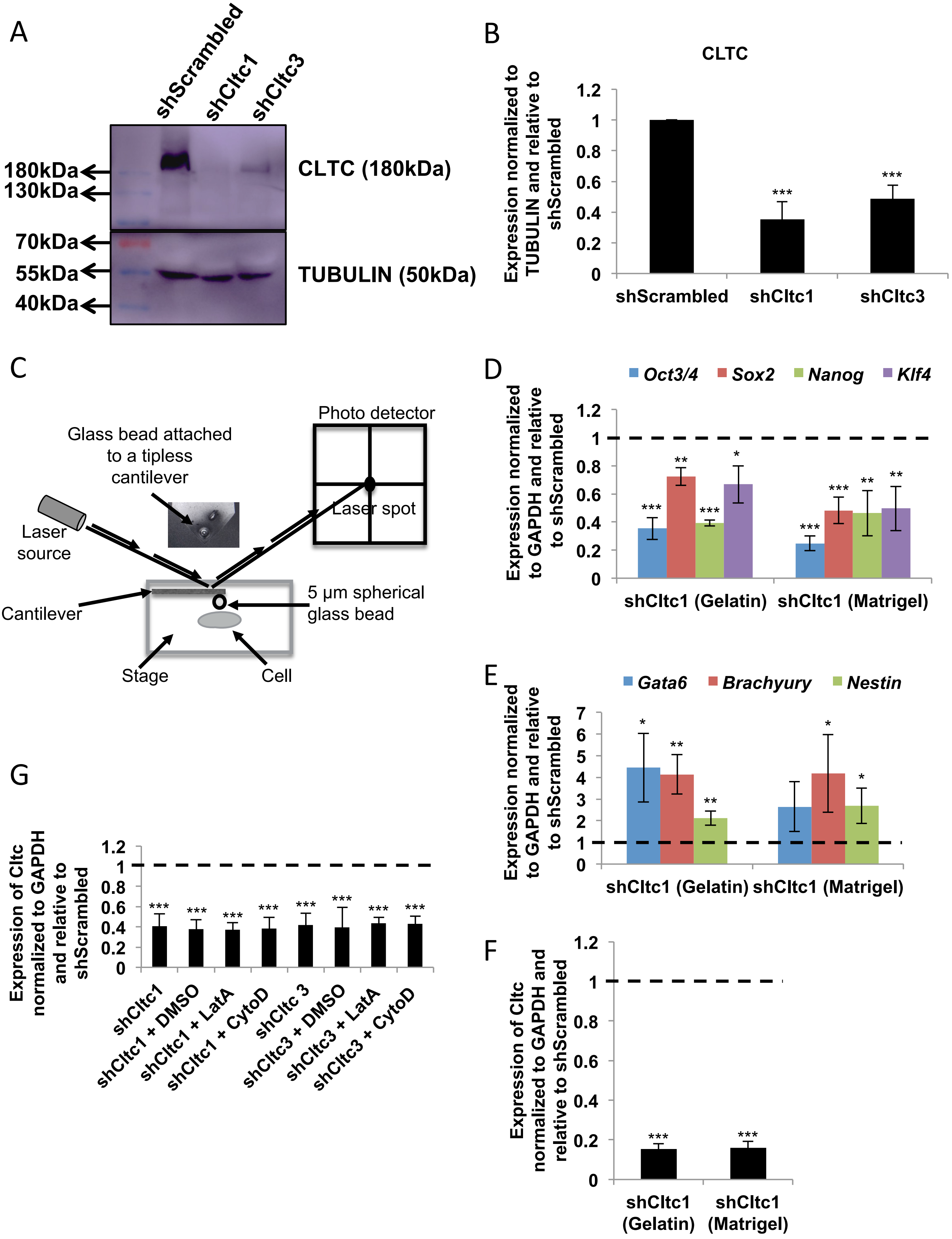

Supplementary Figure 2

A

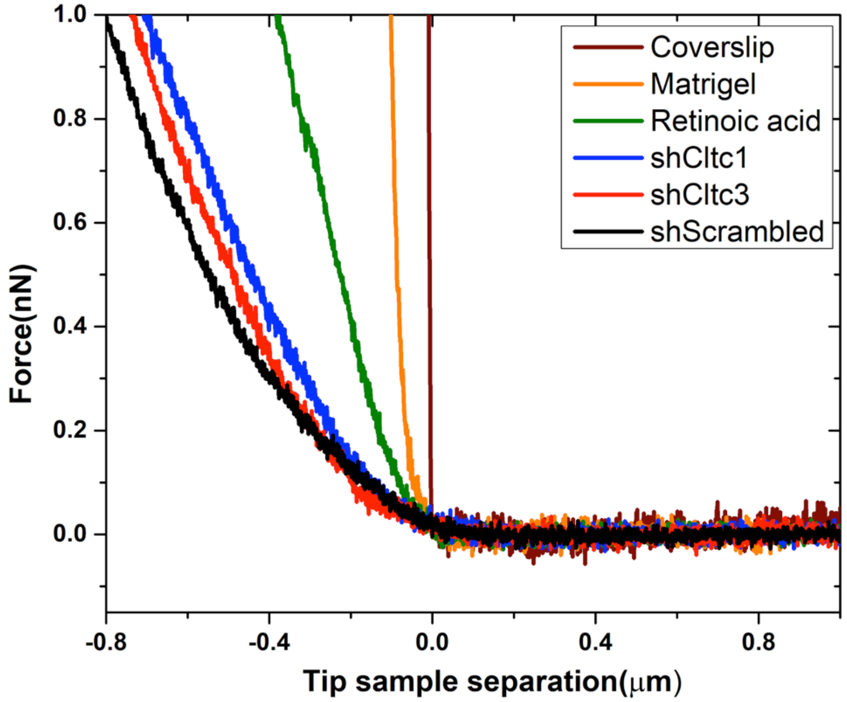

Supplementary Figure 3

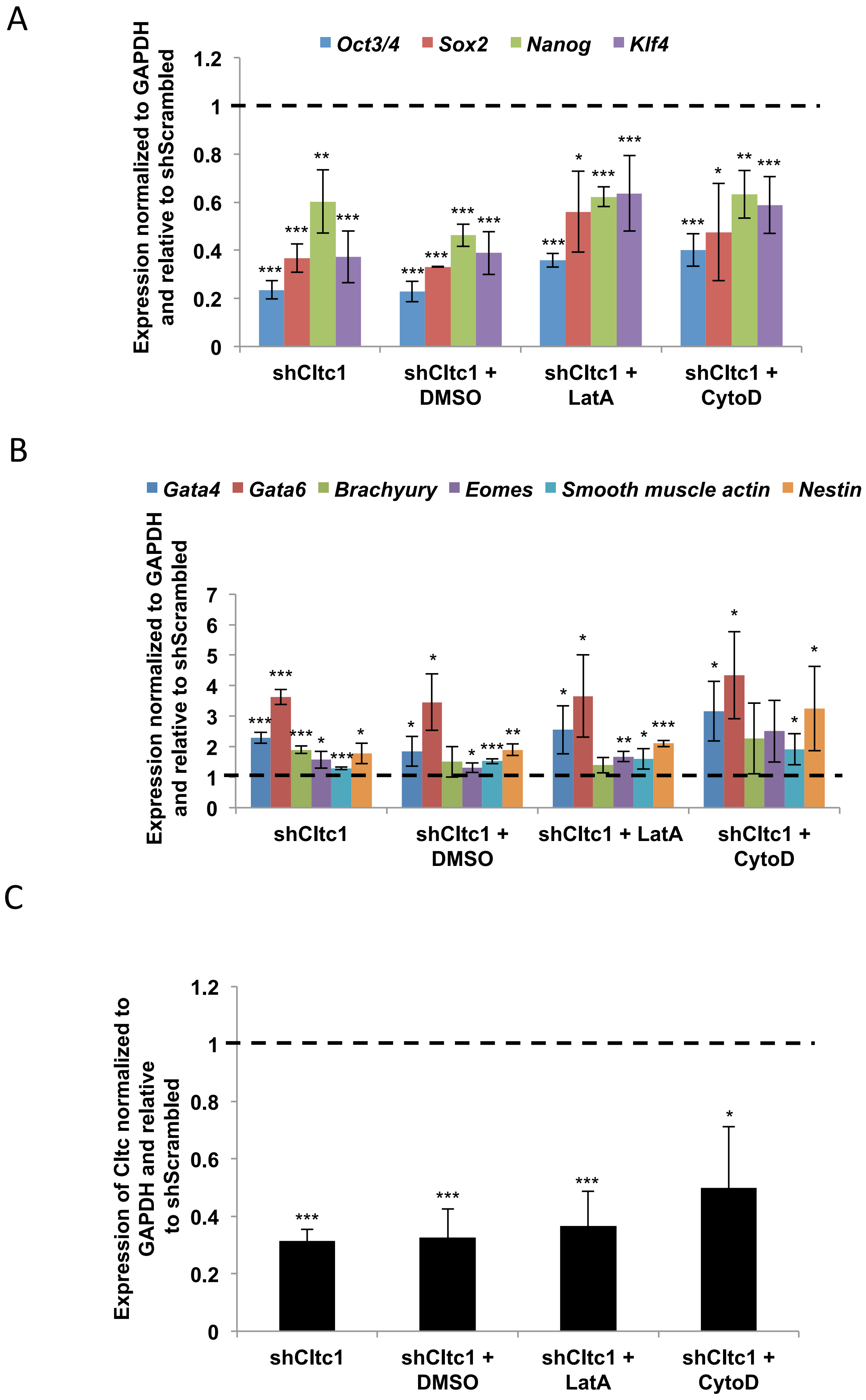

Supplementary Figure 4

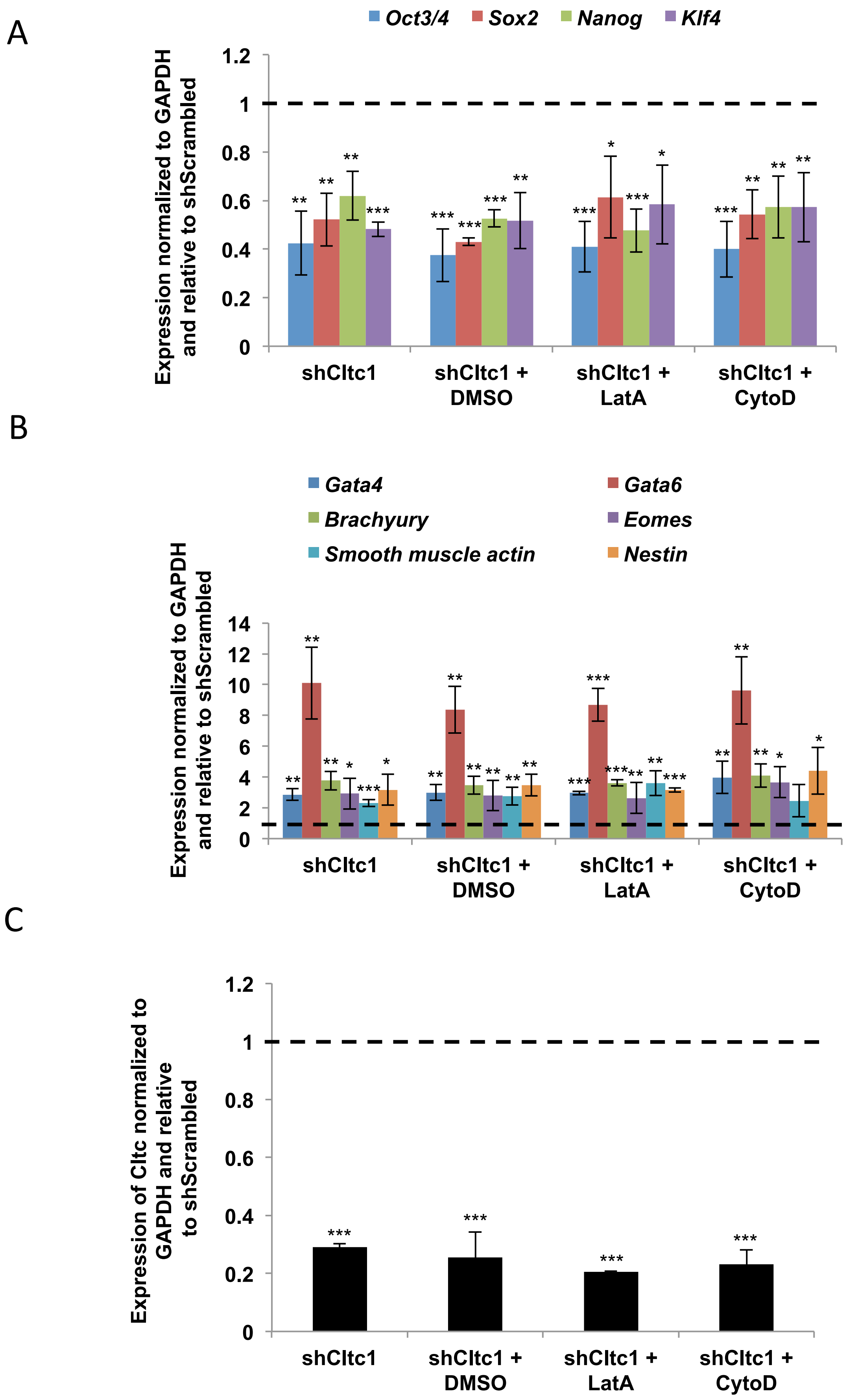

Supplementary Figure 5

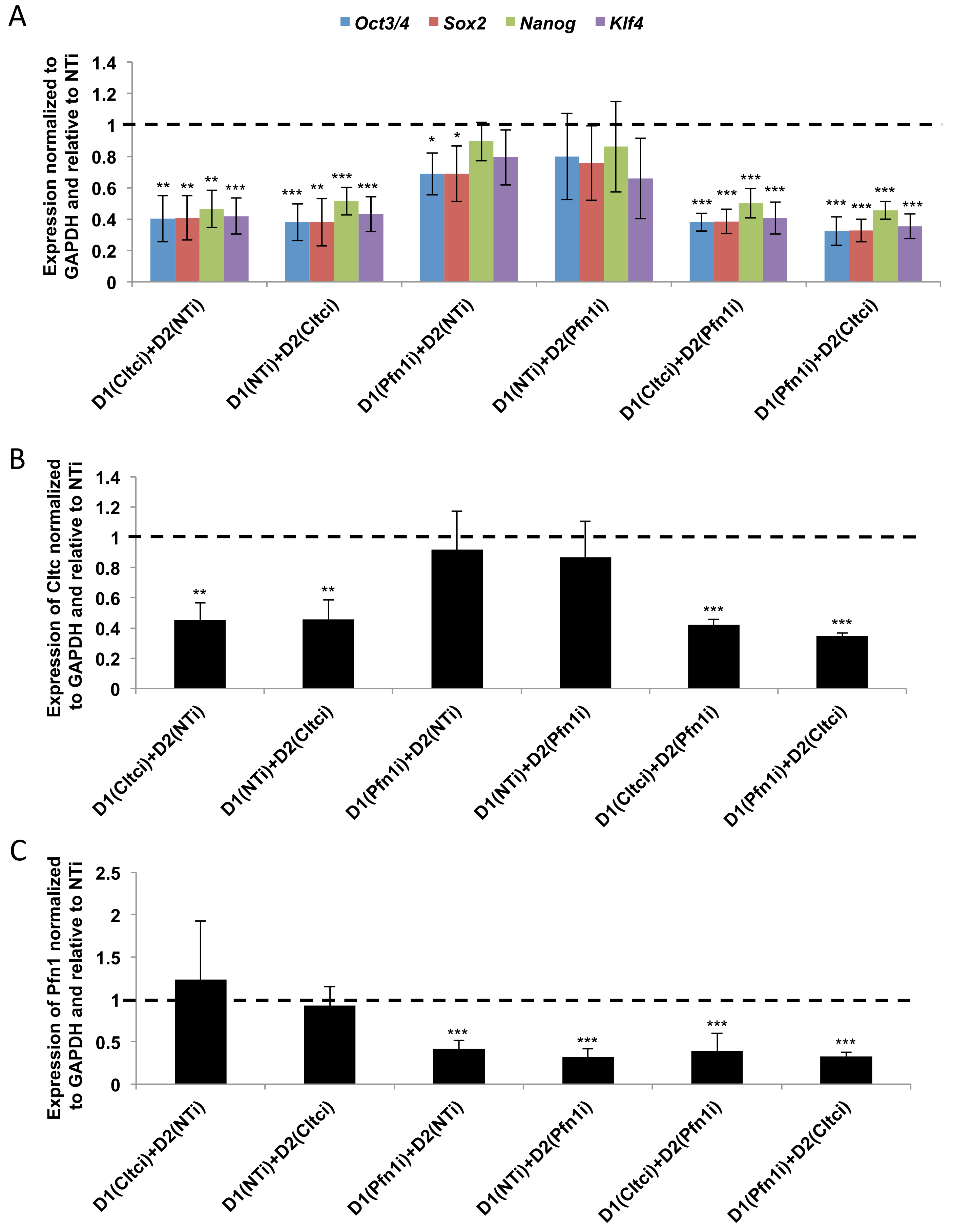

Supplemental Table 1

Sr. no.	Name	shRNA Sequence
1	shCltc1	CCGGCGTGTTCTTGTAACCTTTATTCTCGAGAATAAAGGTTACAAGAACACGTTTTT
3	shCltc3	CCGGCGGTTGCTCTTGTTACGGATACTCGAGTATCCGTAACAAGAGCAACCGTTTTTG

Supplemental Table 2

Sr. no.	Gene	Forward primer 5‘ ⟶ 3’	Reverse primer 5‘ ⟶ 3’
1	*Oct4*	AAAGCCCTGCAGAAGGAGCTAGAA	AACACCTTTCCAAAGAGAACG
2	*Sox2*	ACTTTTGTCCGAGACCGAGAA	CGCGGCCGGTATTTATAATC
3	*Nanog*	GCTCAGCACCAGTGGAGTATCC	TCCAGATGCGTTCACCAGATAG
4	*Klf4*	GACCTCCTGGACCTAGACTTTA	GAAGACGAGGATGAAGCTGAC
5	*Gapdh*	AACAGCAACTCCCACTCTTC	CCTGTTGCTGTAGCCGTATT
6	*Cltc*	GATTTCGCCATGCCCTATT	AGAACAAGCTACAGAGACACAG
7	*Gata4*	CACTTAGGGATATGGGTGTTCC	GGCAGGTGGAGAATAAGGAAG
8	*Gata6*	AGCAAGATGAATGGCCTCAG	CTCACCCTCAGCATTTCTACG
9	*Nestin*	GAAGAGGAGGAGCAGAGAATT	GTGGTCATCGTCTTCCAGATAC
10	*SMA*	GTGCCTATCTATGAGGGCTATG	GGCAGTAGTCACGAAGGAATAG
11	*Eomes*	CCTCCGTACTTGCTTCTACACACTT	AAAGCCTATAGGAACTGTGACATCATAC
12	*Brachyury*	AGGTACCCAGCTCTAAGGAAC	CGAGGCTAGACCAGTTATCATG

Supplemental Table 3

Sr. No.	Antibody name	Catalogue no.
1	Clathrin Heavy Chain (BD Transduction Laboratories)	610500
2	GAPDH (Cell Signaling Technology)	#2118
3	Cofilin (Cell Signaling Technology)	#5175
4	Phospho-Cofilin (Ser3) (Cell Signaling Technology)	#3313
5	Tubulin	T5168
